# Supplementary material for: End Sequence Analysis Toolkit (ESAT) expands the extractable information from single-cell RNA-seq data
Source: Genome Res. 2016 Oct;26(10):1397–410. doi: 10.1101/gr.207902.116 (PMC5052061; doi:10.1101/gr.207902.116)
Supplement: Supplemental Material [file supp_gr.207902.116_Supp_Materials.tar.gz › ESAT-esat_v0.1/doc/html/dir_092d1a752246cbf24923d93621ec8dce.html]

ESAT: java/broad/core/annotation Directory Reference


ESAT


- Main Page
- Related Pages
- Packages
- Classes
- Files

All Classes Namespaces Files Functions Variables Enumerator Pages

- java
- broad
- core
- annotation

annotation Directory Reference

Directory dependency graph for annotation:

|  |  |
| --- | --- |
| Files | |
| file | AnnotationFactory.java |
|  | |
| file | AnnotationFactoryFactory.java |
|  | |
| file | AnnotationHandler.java |
|  | |
| file | AnnotationReader.java |
|  | |
| file | AnnotationReaderFactory.java |
|  | |
| file | AnnotationUtils.java |
|  | |
| file | BasicAnnotationReader.java |
|  | |
| file | BasicGenomicAnnotation.java |
|  | |
| file | BasicGenomicAnnotationReader.java |
|  | |
| file | BasicLightweightAnnotation.java |
|  | |
| file | BasicTwoSubjectAnnotation.java |
|  | |
| file | BED.java |
|  | |
| file | BEDGraph.java |
|  | |
| file | BEDGraphReader.java |
|  | |
| file | BEDReader.java |
|  | |
| file | Feature.java |
|  | |
| file | GenomicAnnotation.java |
|  | |
| file | GenomicAnnotationFilter.java |
|  | |
| file | GFF.java |
|  | |
| file | GFFReader.java |
|  | |
| file | LightweightGenomicAnnotation.java |
|  | |
| file | MaximumContiguousSubsequence.java |
|  | |
| file | RepeatMaskerAnnotation.java |
|  | |
| file | RepeatMaskerAnnotationReader.java |
|  | |
| file | ShortBED.java |
|  | |
| file | ShortBEDReader.java |
|  | |
| file | TwoSubjectAnnotation.java |
|  | |


---

Generated on Fri Aug 15 2014 15:11:15 for ESAT by  

 1.8.7
